# 3,3′-Diindolylmethane Promotes Gastric Cancer Progression *via* β-TrCP-Mediated NF-κB Activation in Gastric Cancer-Derived MSCs

**DOI:** 10.3389/fonc.2021.603533

**Published:** 2021-03-24

**Authors:** Hui Shi, Yaoxiang Sun, Hongru Ruan, Cheng Ji, Jiahui Zhang, Peipei Wu, Linli Li, Chihan Huang, Yuanwang Jia, Xu Zhang, Wenrong Xu, Jiajia Jiang, Hui Qian

**Affiliations:** ^1^ Jiangsu Key Laboratory of Medical Science and Laboratory Medicine, Institute of Stem Cell, School of Medicine, Jiangsu University, Zhenjiang, China; ^2^ Institute of Cancer, Affiliated Aoyang Hospital of Jiangsu University, Zhangjiagang, Suzhou, China; ^3^ Department of Clinical Laboratory, The Affiliated Yixing Hospital of Jiangsu University, Yixing, China; ^4^ Department of Burn Orthopedics, The Affiliated People’s Hospital, Jiangsu University, Zhenjiang, China

**Keywords:** tumor microenvironment, NF- kappa B, GC-MSCs, gastric cancer, 3,3′-diindolylmethane

## Abstract

Gastric cancer is a malignant tumor characterized by high morbidity and invasion. Surgery combined with chemo-radiotherapy is the most common treatment for gastric cancer, while multiple drug resistance always results in treatment failure. Once the anti-tumor drugs enter the tumor foci, tumor cells as well as those found in the microenvironment are affected. However, the effects of drugs on tumor microenvironment (TME) are easily overlooked. In this study, we investigated the effects of the anti-cancer drug 3,3’-diindolylmethane (DIM) on gastric cancer-derived mesenchymal stem cells (GC-MSCs) and their subsequent impact on cancer progression. Surprisingly, we found that the therapeutic concentration of DIM upregulated the expression level of tumor-related factors such as CCL-2, IL-6, and IL-8 in GC-MSCs. The conditioned medium of DIM-treated GC-MSCs promoted the proliferation, invasion, and migration of gastric cancer cells *in vitro* and tumor growth *in vivo*. Mechanistically, DIM enhanced the expression of β-TrCP, an E3 ubiquitin ligase leading to IκBα degradation and NF-κB activation in GC-MSCs. The β-TrCP knockdown partially eliminated positive results caused by DIM. Our results showed that the therapeutic dosage of DIM induced cell death in cancer cells, while enhancing MSC paracrine functions in the stroma to offset the original DIM effect on cancer cells. These findings provide a new mechanism of anti-cancer drug resistance and remind us to adjust the chemotherapeutic scheme by combining the anti-cancer drug with an appropriate signaling pathway inhibitor to block the side effects of drug on targeted TME cells.

## Introduction

Gastric cancer is one of the most common malignancies worldwide and constitutes the second highest morbidity and the third leading cause of cancer-related deaths in China (1, 2). Surgery and chemo-radiotherapy are the primary treatments for gastric cancer. However, these therapeutic regimens are not satisfactory in prolonging patient survival time, mostly due to insufficient pharmaceutical effects and multidrug resistance (3). Scientists have put considerable effort into determining the mechanisms of drug resistance in gastric cancer. Previous research suggests that drug degradation, anti-apoptosis, immune escape, epithelial-mesenchymal transition (EMT), cell stemness, autophagy, epigenetic modifications, and upregulation of multidrug resistance (MDR)-related genes are all involved in the potential risk of treatment failure of gastric cancer (4). Current research has mainly focused on the intrinsic or acquired drug resistance in gastric cancer cells; however, once drugs enter the tumor foci, both tumor cells and those in the tumor microenvironment (TME) are affected. Nevertheless, little attention has been paid to the influence of chemotherapeutic drugs on the TME and the subsequent feedback effect on the cancer cells.

TME is vital to the growth of cancer cells because it provides the necessary support and nutrition as in the relationship between soil and seeds (5). TME contains matrix cells, vascular endothelial  cells, and immune cells in addition to tumor cells, with major characteristics including low oxygen, low pH, and high levels of proteolytic enzymes and cytokines. These particular features and peculiarity provide opportunities for tumor proliferation, invasion, and migration (6). Mesenchymal stem cells (MSCs) exist in TME and facilitate tumor progression by secreting various cytokines, suppressing immune responses, and remodeling the extracellular matrix of tumors. Houghton et al. (7) found that the fusion of MSCs and gastric epithelial cells under *Helicobacter  pylori* infection can induce gastric cancer formation. Thus, alterations in the external environment, such as the treatment of chemotherapeutic drugs on MSCs, may influence its primary impact on tumor progression. We previously isolated and identified MSCs from gastric cancer (GC-MSCs) for the first time (8), and proved that GC-MSCs could prompt gastric cancer metastasis *via* EMT induction (9) and serve as a potential target for gastric tumor treatment (10). Thus, we hypothesized that alterations in the external environment as the treatment of chemotherapeutic drugs may influence the function of MSCs on gastric cancer progression. In this study, we aimed to investigate the relationship between the anti-cancer drug 3,3-diindolylmethane (DIM), GC-MSCs, and gastric cancer progression.

DIM is a small-molecule compound and a major active metabolite of indole-3-carbinol, which can be extracted from cruciferous vegetables. Many studies have shown that DIM can inhibit proliferation and induce apoptosis in various cancer types (11). Previously, we found that low levels of DIM activated Wnt4 autocrine signaling to enhance the progression of gastric cancer cells (12). Moreover, our research also indicated that DIM could promote the stemness of human umbilical cord-derived mesenchymal stem cells (hucMSCs) by increasing exosome mediated Wnt11 autocrine signaling (13), so that the stemness-enhanced hucMSCs could be used in tissue regeneration. However, the effects of DIM on TME-derived MSCs and their subsequent influence on tumors remains unknown.

In this study, we treated GC-MSCs with the regular dosage of DIM (depending on IC_50_) and found that GC-MSCs expressed a high level of oncogenic factors such as CCL-2, IL-6, IL-8, and TGF-β. Furthermore, this expression was triggered by the activation of β-TrCP/NF-κB signaling pathway. The conditioned medium of GC-MSCs pretreated with DIM could promote proliferation, invasion, and migration of gastric cancer cells. β-TrCP knockdown eliminated positive results caused by DIM. Collectively, the therapeutic dosage of DIM could induce cancer cell death, while enhancing MSC paracrine functions in the stroma to offset the cell death induction, which provides a new vision on the application of anti-tumor drugs. A chemotherapeutic scheme that combines the use of a signaling pathway inhibitor to block the side effect from drug-targeted TME cells could be considered.

## Materials and Methods

The study was approved by the Medical Ethics Committee and Ethics Committee for Experimental Animals of Jiangsu University (IRB protocol number: 2020161).

### Cell Culture, GC-MSC Isolation and Identification

Human gastric cancer cell lines HGC-27, SGC7901, and MGC-803 were purchased from the Institute of Biochemistry and Cell Biology at the Chinese Academy of Sciences (Shanghai, China). Cells were cultured in high-glucose Dulbecco’s modified Eagle medium (DMEM) (Gibco, Grand Island, NY, USA) containing 10% fetal bovine serum (FBS; Gibco, USA). Cells were cultured at 37°C in humidified air with 5% CO_2_. HucMSCs were isolated as previously described (14) and maintained in low-glucose DMEM (Gibco, Grand Island, NY, USA) containing 10% FBS.

The gastric cancer tissues were obtained from patients with gastric adenocarcinoma in The Affiliated People’s Hospital of Jiangsu University, Zhenjiang, China. Fresh, sterile gastric carcinoma tissue specimens were collected during surgery. The specimens were immersed in 95% ethanol for 2-3 sec to avoid contamination, and then washed with PBS and antibiotics several times to remove the blood. The surface of the cancer tissues was removed and the inner parts were cut into 1- to 3-mm^3^-sized pieces and floated in Dulbecco’s modified Eagle’s medium with low glucose (LG-DMEM) (Gibco, USA), containing 10% fetal bovine serum (FBS, Gibco, USA), penicillin (100 U/ml) and streptomycin (100 lg/ml). The pieces of cancer tissues were subsequently incubated at 37°C in humidified air with 5% CO2. After culturing for 15 days, colonies of fibroblast-like cells appeared. When their confluens reached 80%, the cells were harvested by 0.25% trypsin-1 mM EDTA and re-plated into larger culture flasks at a 1:3 split ratio. These gastric cancer-derived MSC-like cells at passage four were used for subsequent experiments. As for the identification of GC-MSCs, the expression of specific surface antigens CD44 (BD Pharmingen), CD105 (Miltenyi), CD34 (BD Pharmingen), CD45 (BD Pharmingen) of GC-MSCs was detected by flow cytometry, and multi-directional differentiation potential was assessed through osteogenic and adipogenic differentiation assays according to the manufacturer’s instructions (Cyagen). Cells were stained with alizarin red and Oil-Red-O (for lipid droplets) on Day 14.

### Conditioned Medium Preparation

GC-MSCs were propagated in Dulbecco’s modified Eagle’s medium with low glucose (LG-DMEM) (Gibco, USA) containing 10% FBS (Gibco, USA) and used for subsequent experiments at passage four. GC-MSCs were treated with DMSO or DIM 50 μM for 48 h, then the cell supernatant was discarded and washed with PBS for three times (referred as the final eluant) and replenished with fresh culture medium. After another 48 h, the cell supernatant was collected as the conditioned medium (CM). The gastric cancer cells SGC-7901, MGC80-3, HGC-27 were treated with the CM (CM: LG-DMEM=1:1) for 48 h.

### Colony Formation Assay

Gastric cancer cells SGC-7901, MGC80-3 and HGC-27 were treated with CM from GC-MSC mentioned above for 48 h, harvested, and seeded into 35-mm plates (1000 cells/well) overnight under standard conditions for 10 days. The medium containing was changed at 3-day intervals. At the end of the incubation period, the cultures were fixed with 4% paraformaldehyde and stained with crystal violet.

### MTT Assay

MTT assay was used to determine the viability of different cells (HGC-27, SGC-7901, hucMSC, and GC-MSC) treated with different concentrations of DIM. Cells were inoculated into 96-well plates at a density of 1.5×10^4^ cells/well. After 24 h incubation, the cells were treated with DIM at concentrations of 1, 10, 25, 50, 100, 200, 300, and 400 μM DIM or the same volume of DMSO (0 μM). After 24 h incubation, 20 μL of MTT (0.5 mg/mL) was added to each well and cultured for 4 h. The formazan crystals formed were solubilized in DMSO, and the absorbance of each well was read in a microplate reader at a wavelength of 490 nm.

### RNA Extraction, RT-PCR, and Real-Time RT-PCR

Total RNA was extracted from cells and tissues using TRIZOL Reagent (Invitrogen, Carlsbad, CA, USA) according to the manufacturer’s instructions, and equal amounts of RNA were used for RT-PCR and real-time RT-PCR analyses. β-actin was used as an internal control. The sequences of specific primers are listed below:

*TGF*-β, 5′-CAGAGGTGGTGGGGTAGAGA-3′ and 5′-CATTGCCACTCACAATGTCC-3′;
CCL-2, 5’-GAACCGAGAGGCTGAGACTA-3’and 5’-GCCTCTGCACTGAGATCTTC-3’;
IL-6, 5’-ACATCCTCGACGGCATCTC-3’ and 5’-AGCTCTGGCTTGTTCCTCAC-3’;
IL-8, 5’-GCTCTGTGTGAAGGTGCAGTTT-3’ and 5’-TTCTGTGTTGGCGCAGTGT-3’;
β-TrCP, 5’-CAGTTCTGCACTTGCGTTTC-3’ and 5’-CTCACTACCAGCCTGTCCCT-3’;
β-actin, 5’-CACGAAACTACCTTCAACTCC-3’ and 5’-CATACTCCTGCTTGCTGATC-3’.

### RNA Inference

Specific siRNA against β-TrCP was produced by GenePharma (Suzhou, Jiangsu, China). The sequence of β-TrCP siRNA was *5’-GAGAGAGAAG ACUGUAAUAdTdT-3’*. GC-MSCs (1×10^6^ cells/well) were grown in six-well plates and transfected with siRNAs using LipoFiter transfection reagent (Hanbio, Shanghai, China) for 6 h. Following 24 h incubation, the transfected cells were harvested in 10 cm plates. The conditioned medium and cells were collected for subsequent experiments.

### Transwell Migration Assay

Gastric cancer cells SGC-7901, MGC80-3 and HGC-27 were treated with CM from GC-MSC mentioned above for 48 h, harvested, and seeded into the top chamber, and 10% FBS-containing medium was placed into the bottom chamber. After incubation at 37°C in 5% CO_2_ for 12 h, the cells remaining on the upper surface of the membrane were removed with a cotton swab. The cells that migrated through the 8-μm sized pores and adhered to the lower surface of the membrane were fixed with 4% paraformaldehyde, stained with crystal violet, and imaged.

### Wound Healing Assay

Gastric cancer cells SGC-7901, MGC80-3 and HGC-27 were pretreated with CM from GC-MSC mentioned above for 48 h, harvested, and seeded at a density of 2×10^5^ cells/well in six-well plates and incubated at 37°C in 5% CO_2_ for 24 h to create confluent monolayers. The monolayers were scratched with a sterile pipette tip. To measure cell mobility, images from five random fields at 24 h after scratching were obtained. The width of the original scratch was measured using the NIH ImageJ image processing software (http://rsb.info.nih.gov/nih-image/). The migration ratio was calculated as follows: (the width of the original scratch-the width of the actual scratch)/the width of the original scratch×100.

### Western Blot Analysis

Pretreated GC-MSCs were homogenized and lysed in RIPA buffer supplemented with proteinase inhibitors. Equal amounts of a total protein were loaded and separated on a 12% sodium dodecyl sulfate-polyacrylamide gel electrophoresis (SDS-PAGE) gel. Following electrophoresis, the proteins were transferred to a polyvinylidene difluoride membrane, blocked in 5% (w/v) fat-free milk, and incubated with the primary antibodies. The primary antibodies used were: β-TrCP (CST, USA); p-NFκB (CST, USA); t-NFκB (CST, USA); IκBα (CST, USA); GAPDH (CWBIO, China); goat anti-rabbit IgG-HRP, and goat anti-mouse IgG-HRP (CWBIO, China).

### Xenograft Mouse Model

Twenty male BALB/c nu/nu mice (Laboratory Animal Center of Shanghai, Academy of Science, Shanghai, China) aged 4-6 weeks were randomly divided into two groups (five mice/group). MGC80-3 cells were pretreated with different types of conditioned medium of GC-MSCs for 48 h, then 2.5×10^6^ cells in 200 μl PBS were implanted subcutaneously into the right flanks of the mice. The mice were fed normally and the tumors were harvested 30 days after the implantation. Tumor size and weight were measured.

### Apoptosis Assays

GC cells MGC-7901 were pretreated with different types of conditioned medium of GC-MSCs for 48 h, with PBS used as a control, and then cells were collected and seeded at a density of 2×10^5^ cells/well in six-well plates and incubated at 37°C in 5% CO_2_ for 24 h, then treated with 50 μM DIM for 48 h. Annexin V/PI staining of these cells was performed to detect apoptosis. In brief, cells were collected and stained with Annexin V/PI for 15 and 30 min, then the multicolor flow cytometry was performed according to standard protocols (FACS Canto II, BD Biosciences). The results were analyzed using CellQuest software.

### Statistical Analysis

Data are expressed as mean ± SD. The statistical significance of differences between two groups was determined using two-tailed Student’s *t*-test. The significance of differences among multiple groups was determined using one-way ANOVA. All experiments were performed at least in triplicates (n=3). *P* < 0.05 was considered statistically significant. All statistical analyses were performed using GraphPad Prism (GraphPad Software, La Jolla, CA).

## Results

### DIM Increases the Expression of Tumor-Related Factors in GC-MSCs

**Figure 1:**
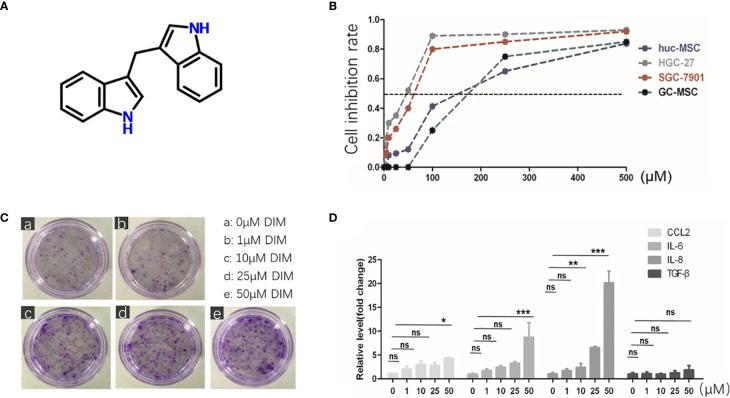
Regular dose of DIM promotes tumor progression in GC-MSCs. **(A)** Chemical structure of DIM. **(B)** Non-toxic concentrations for the subsequent experiments in GC-MSCs and gastric cancer cell lines (SGC7901 and HGC-27) are selected by MTT assay. Gastric cancer cell lines, hucMSCs, and GC-MSCs are treated with 500, 250,100, 50, 25, 10, and 5 μM of DIM or the same volume of DMSO as control (0 μM DIM). **(C)** Representative images of colony formation in GC-MSCs treated with 1, 10, 25, and 50 μM DIM or the same volume of DMSO (0 μM DIM) for 48 h. Original magnification, 40 X. **(D)** Expression of *CCL2*, *IL-6*, *IL-8*, and *TGF-β* genes in GC-MSCs treated with 1, 10, 25, and 50 μM DIM or the same volume of DMSO (0 μM DIM) for 48 h were detected using real-time RT-PCR. **(E)** is moved to **Figure S2** **(A)**.

DIM is a natural compound harvested from cruciferous vegetables, which belongs to the class of indole glucosinolates with the molecular formula C17H14N2 (**Figure 1A**). To explore its influence on GC-MSCs, we firstly isolated and identified GC-MSCs by verifying their adipogenesis and osteogenesis capacity in inducing reagent as well as the representative markers (**Figures S1A, B**). We used the MTT assay to determine the optimum concentration of DIM required to induce cell death. We treated gastric cancer cell lines (SGC-7901 and HGC-27) and GC-MSCs with different DIM concentrations and measured cell proliferation rate. The results showed that 50 μM DIM induced cell death in gastric cancer cells (SGC-7901 IC_50 =_ 60.743 μM, HGC-27 IC_50 =_ 42.812 μM), resulted in partial inhibition of hucMSCs (IC_50 =_ 158.11 μM), and had no cytotoxic effect on GC-MSCs (**Figure 1B**). To further confirm the effects of DIM on GC-MSCs, we analyzed the colony formation ability in GC-MSCs pretreated with different concentrations of DIM (0, 1, 10, 25, and 50μM DIM) for 48 h. Ten days later, we found that the anti-cancer dosage of DIM not only had no inhibitory effect on GC-MSCs but promoted cell proliferation, especially at 50 μM (**Figure 1C**). Based on these results, we evaluated the expression as well as the secretion level of inflammatory cytokines (CCL-2, IL-6, IL-8, TGF-β) in GC-MSCs pretreated with different concentrations of DIM. The data showed that 50 μM DIM significantly increased the expression of CCL-2, IL-6, and IL-8 (**Figure 1D**; **Figure S4**). However, a higher concentration of 100 μM reversed these results (**Figure S2A**). Thus, we hypothesized that GC-MSCs pretreated with DIM may influence the progression of gastric cancer by secreting pro-inflammatory cytokines.

### Conditioned Medium of GC-MSCs Treated With DIM Promotes Gastric Cancer Cell Development

**Figure 2:**
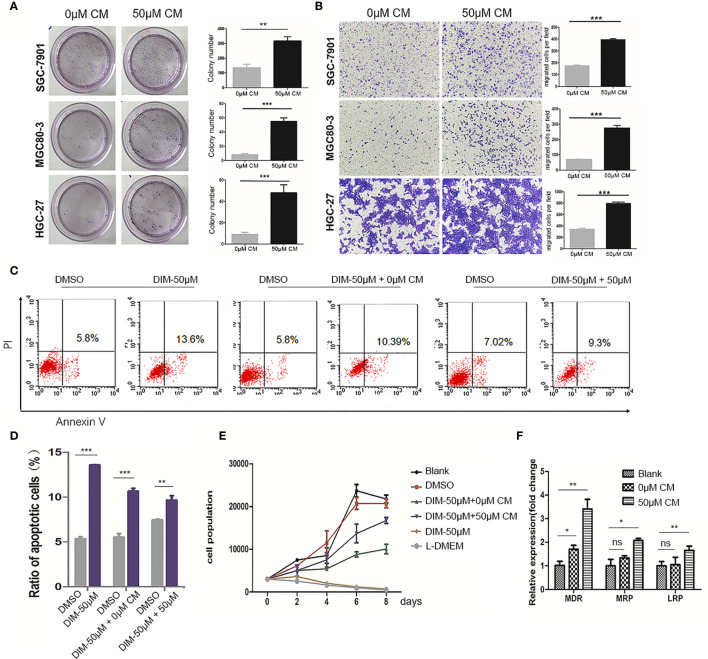
Conditioned medium (CM) of GC-MSCs treated with DIM (50 μM for 48 h) promoted gastric cancer progression. CM of GC-MSCs pre-treated with 50 μM (50 μM-CM) or the same volume of DMSO (0 μM-CM) for 48 h is collected. **(A)** SGC-7901, MGC80-3, and HGC-27 cells are co-cultured with CM of GC-MSCs for 48 h, and then collected for colony formation assay for 7 days, Original magnification, 40 X. **(B)** The migratory ability of SGC-7901, MGC80-3, and HGC-27 cells treated with the CM of GC-MSCs is evaluated using trans-well migration assay. Original magnification, 100 X. **(C, D)** MGC80-3 are pretreated with CM of GC-MSCs for 48 h respectively, with PBS used as a control and then treated with 50 μM DIM for 48 h. Cells are collected and labeled with Annexin V/PI and detected using flow cytometry. **(E)** MGC80-3 are pre-treated with CM of GC-MSCs for 48 h and treated with 50 μM DIM, then the growth curve is compared with these in MSC80-3 treated 50 μM DIM or the same volume of PBS, or cultured with the L-DMEM. **(F)** MGC80-3 pretreated with CM of GC-MSCs, or the same volume of PBS. Real-time RT-PCR detects the expression of drug resistance genes *MDR*, *MRP*, and *LRP*. (***P < 0.001 compared with the control group).

To verify our hypothesis, we collected the conditioned medium (CM) of GC-MSCs that were pretreated with 50 μM of DIM (50 μM-CM) or DMSO (0 μM-CM) as a solvent control for 48 h, and then treated gastric cancer cells (SGC-7901, MGC80-3, and HGC-27) for 48 h. Subsequently, colony formation and transwell migration assays were performed to observe alterations in gastric cancer cells. The treatment with 50 μM-CM promoted proliferation of SGC-7901, MGC80-3, and HGC-27 cells; the number of colonies were significantly increased (**Figure 2A**). The migration rates of gastric cancer cells in the 50μM-CM group were much higher than those in the control group (**Figure 2B**). The application of DIM did induce a partial death in cancer cells, while interestingly, we found that DIM-treated GC-MSC-derived CM showed a protective effect rescuing tumor cells from apoptosis (**Figures 2C, D**). To further confirm the effect of CM derived from GC-MSCs on gastric cancer cells under the DIM treatment, we measured the growth curves of MGC80-3 co-cultured with 0 μM-CM or 50 μM-CM for 48 h together with DIM treatment and compared them with growth curves of MGC80-3 and MGC80-3 treated with 50 μM DIM (the GC-MSCs were cultured in L-DMEM, L-DMEM as a control). We found that L-DMEM and 50 μM DIM fully inhibited the growth of MGC80-3, while 0 μM-CM of GC-MSCs protected MGC80-3 from DIM and 50 μM-CM enhanced this function (**Figure 2E**). We also detected the expression of drug resistance genes MDR, MRP, and LRP in both MGC80-3 and MGC80-3 pretreated with CM of GC-MSCs. The levels of MDR, MRP, and LRP were significantly increased in MGC80-3 treated with 50 μM-CM (**Figure 2F**). These data implied that the CM of GC-MSCs (especially CM of DIM-treated GC-MSCs) could promote the development of gastric cancer and protect gastric cancer cells from the lethal effect of DIM. This phenomenon was enhanced by the increase in the expression of drug resistance genes in gastric cancer cells treated with 50 μM-CM, which means that the tumor microenvironment was rather complicated that DIM did not just kill tumor cells, but also functions on GC-MSCs to promoted tumor progression. The above results showed that GC-MSCs in TME might have a side effect on anti-cancer treatment.

### DIM Promotes Gastric Cancer Progression by Activating the NF-κB Signaling in GC-MSCs

**Figure 3:**
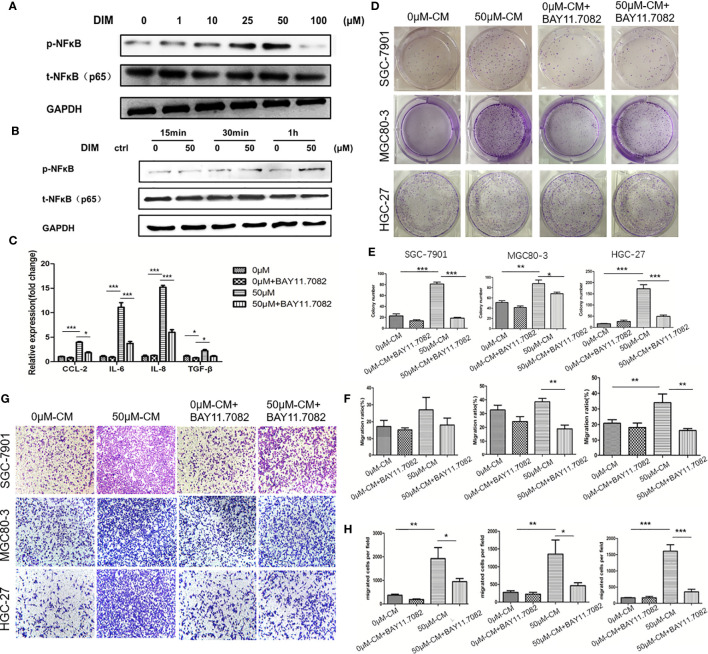
Regular dosage of DIM promotes the activation of the NFκB signaling pathway in GC-MSCs, which contributes to gastric cancer progression promoted by the GC-MSCs conditioned medium (CM). **(A)** Western blot detects the expression of p-NFκB in GC-MSCs treated with 1, 10, 25, 50, and 100 μM DIM or the same volume of DMSO (0 μM) for 48 h. **(B)** Western blot detects the expression of p-NFκB in GC-MSCs treated with 50 μM DIM for 15 min, 30 min or 1 h. **(C)** GC-MSCs treated with 50 μM DIM and with or without NFκB signaling pathway inhibitor BAY 11.7082 for 48 h, GC-MSCs treated with the same volume of DMSO and with or without BAY 11.7082 for 48 h as a control. Real-time RT-PCR detects the expression of *CCL*-2, *IL-6*, *IL-8*, and *TGF-β*. GC-MSCs are treated with DMSO, DMSO+ BAY 11.7082, 50 μM DIM, or 50 μM DIM + BAY 11.7082. CM is collected and cultured with of SGC-7901, MGC80-3, and HGC-27 cells respectively. Colony formation assay **(D, E)**, transwell migration assay **(G, H)**, and wound healing assays **(F)** are used to determine the proliferation and migration abilities of SGC-7901, MGC80-3, and HGC-27 cells in different groups (n = 3, *P < 0.05, **P < 0.01, ***P < 0.001 compared with the control group).

To further determine the mechanism by which DIM-pretreated GC-MSCs promoted gastric cancer progression, we detected the level of pNF-κB in GC-MSCs pretreated with different concentrations of DIM for 48 h (1, 10, 25, 50, and 100 μM of DIM or the same volume of DMSO) by using western blot analysis. The results showed that DIM increased the expression of p-NFκB in GC-MSCs in a concentration dependent manner within the 50 μM range but decreased its expression at 100 μM after 1 h of DIM treatment (**Figures 3A, B** **;** **Figure S3**). To confirm these results, we used BAY 11.7082 to inhibit the NF-κB signaling pathway in GC-MSCs treated with 50 μM DIM and DMSO (0 μM). The results showed that the expression levels of CCL-2, IL-6, IL-8 were decreased in BAY 11.7082 group (**Figure 3C**). We then collected the CM of GC-MSCs treated with 0 μM, 50 μM, 0 μM of DIM + BAY 11.7082, and 50 μM DIM + BAY 11.7082 for 48 h, to treat gastric cancer cells (SGC-7901, MGC80-3, and HGC-27). We found that 50μM-CM promoted proliferation (**Figures 3D, E**) and migration capacity (**Figures 3F–H**) in gastric cancer cells, and these effects were inhibited by the NF-κB signaling pathway inhibitor BAY11.7082. These results confirmed that the anti-cancer dosage of DIM induced the activation of the NF-κB signaling pathway in GC-MSCs to secrete tumor-related factors CCL-2, IL-6, and IL-8 and promote the progression of gastric cancer cells.

### DIM Increases the Expression of NF-κB in GC-MSCs by β-TrCP-Mediated IκBα Inhibition

**Figure 4:**
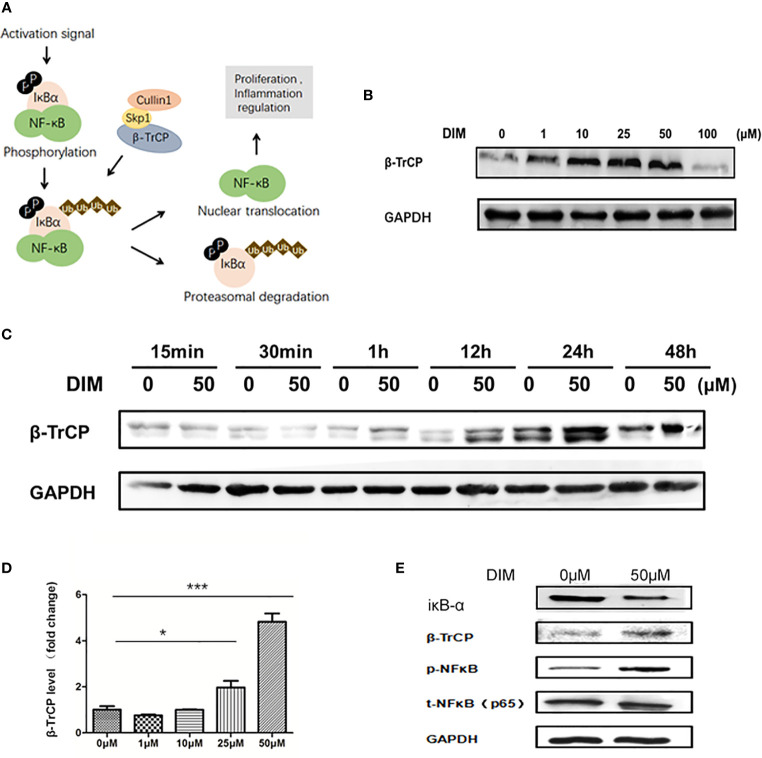
DIM increases the expression of NFκB in GC-MSCs by inhibiting β-TrCP. **(A)** Schematic diagram of the βTRCP-NFκB signaling pathway. **(B)** Western blot analysis of the expression of β-TrCP in GC-MSCs pretreated with 1, 10, 25, 50, and 100 μM DIM or the same volume of DMSO (0 μM) for 48 h. **(C)** The level of β-TrCP in GC-MSCs treated with 50 μM DIM or the same volume of DMSO (0 μM) for 15 min, 30 min, 1 h, 12 h, 24 h, or 48 h are determined using Western blot. **(D)** Real-time RT-PCR is used to detect the *β-TrCP* gene in GC-MSCs pretreated with 1, 10, 25, and 50 μM DIM or the same volume of DMSO (0 μM) for 48 h. **(E)** The expression of IκBα, β-TrCP, and p-NF-κB in GC-MSCs pretreated with 50 μM DIM or the same volume of DMSO (0 μM) for 48 h are detected using western blotting (n = 3, *P < 0.05, **P < 0.01, ***P < 0.001 compared with the control group).

The activation of NF-κB signaling pathway is regulated by IκBα, which is the substrate of E3 ubiquitin ligase β-TrCP (**Figure 4A**). We used western blot analysis to detect the variations in β-TrCP in GC-MSCs pretreated with different concentrations of DIM (1, 10, 25, 50, and 100 μM and the same volume of DMSO as control) for 48 h. The expression of β-TrCP was increased in a concentration-dependent manner within the range of 50 μM of DIM and was decreased at 100 μM (**Figure 4B**), consistent with the variations in p-NFκB level. The levels of the *β-TRCP* gene in GC-MSCs above were similar to the observed protein expression (**Figure 4D**). This phenomenon was also observed just after GC-MSCs were treated with DIM for 1 h and exited for quite a long time as well as p-NFκB (**Figure 4C**). Based on these results, we chose 50 μM DIM to pretreat GC-MSCs for 48 h and determined the expression of IκBα, β-TrCP, p-NFκB, using DMSO as the control. DIM (50 μM) increased the expression of β-TrCP and inhibited the expression of IκBα, decreasing the degradation of p-NFκB (**Figure 4E**). These results showed that β-TrCP was the regulatory molecule of the NF-κB signaling pathway in DIM-treated GC-MSCs.

### Knock Down of β-TrCP in GC-MSCs Weakens Their Tumor Progression Effect on Gastric Cancer Cells

**Figure 5:**
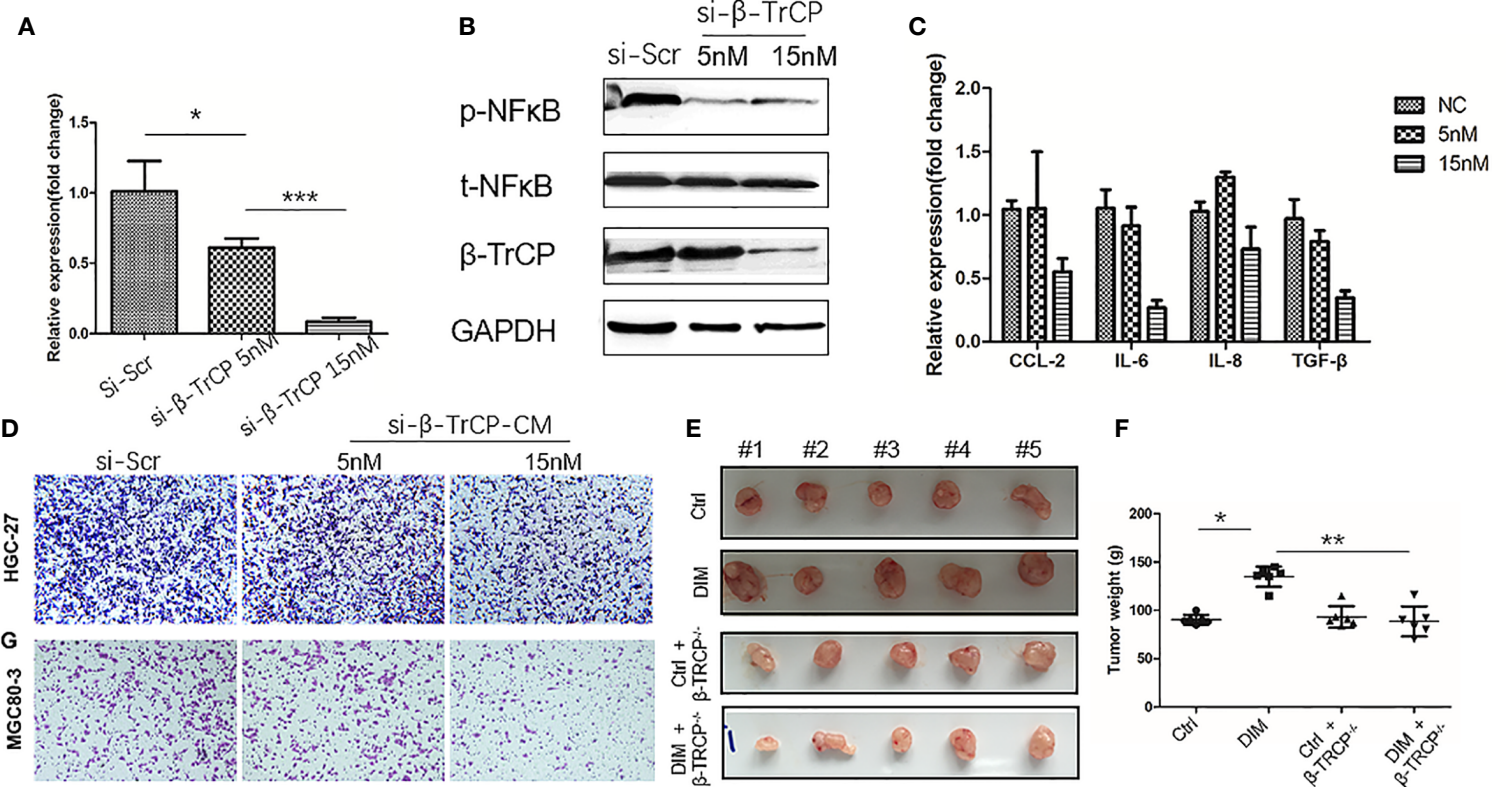
Knock-down of β-TrCP in GC-MSCs weakens the tumor progression effect on gastric cancer cells by the conditioned medium (CM) of DIM-pretreated GC-MSCs. **(A)** Real-time RT-PCR are used to determine the level of *β-TRCP* in GC-MSCs after transfection with different concentrations (NC, 5 nM, 15 nM) of *β-TRCP*-siRNA. **(B)** Western blot detects the expression of β-TrCP, p-NFκB in GC-MSCs transfected by *β-TrCP*-siRNA (NC, 5 nM, 15 nM). **(C)** The level of *CCL2*, *IL-6*, *IL-8*, and *TGF-β* genes in GC-MSC transfected with *β-TrCP*-siRNA (NC, 5, 15 nM) are monitored with Real-time RT-PCR. GC-MSCs are transfected with *β-TrCP*-siRNA (NC, 5, 15 nM) and pretreated by 50 μM DIM for 48 h, and CM is collected, **(D)** Migration ability of HGC-27 and MGC80-3 co-cultured with different CM are determined using transwell migration assay. **(E)** MGC80-3 are pre-cultured with CM of either GC-MSCs (ctrl), 50 μM DIM-treated GC-MSCs for 48 h (DIM), GC-MSCs transfected with *β-TrCP*-siRNA (ctrl+βTRCP-/-), 50μM DIM-treated GC-MSCs transfected with *β-TrCP*-siRNA (DIM+ β-TrCP-/-), subcutaneously injected into mice, and the images of the excised tumors at 30 days post-inoculation are taken. **(F)** Tumor weight is evaluated in mice transplanted with MGC80-3 in **(E)**. (*P < 0.05, **P < 0.01 compared with the control group).

**Figure 6:**
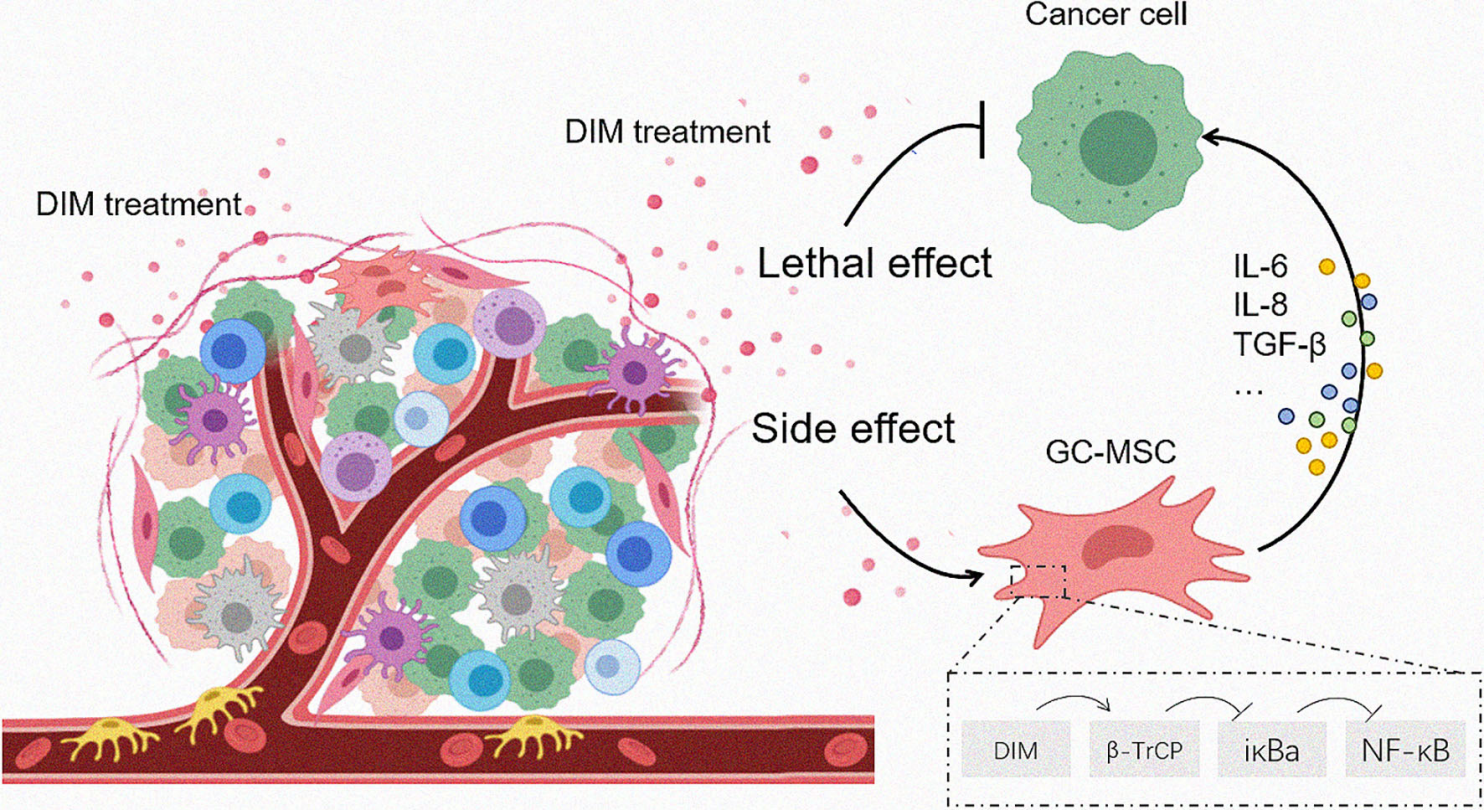
Proposed model for DIM-induced GC-MSC stimulation in the TME and the subsequent effects on gastric cancer cells. Once the anti-tumor drugs enter the tumor foci, both tumor cells as well as the cells in the TME are affected. The effective dose of DIM induces cell death in tumor cells while reversely upregulates the expression of CCL-2, IL-6, IL-8 in GC-MSCs by β-TrCP-mediated IκBα degradation and NFκB activation, so that to enhance the tumor progression as a side effect.

To further confirm that DIM treatment activated NF-κB pathway in GC-MSCs by upregulating the expression of β-TrCP, we knocked down β-TrCP in GC-MSCs. Different concentrations of *β-TrCP* siRNA (5 and 15 nM) were transfected into GC-MSCs. The level of *β-TrCP* mRNA was decreased in a concentration dependent manner (**Figure 5A**). The protein levels of β-TRCP and p-NFκB in GC-MSCs treated with β-TrCP siRNA (5n M, 15 nM) were also reduced (**Figure 5B**). Real-time PCR was used to detect the expression of *CCL-2*, *IL-6*, and *IL-8* genes in GC-MSCs treated with *β-TrCP* siRNA, and the levels of these cytokines were decreased in response to the siRNA concentration gradient (**Figure 5C**). Subsequently, we treated the GC-MSCs and β-TrCP^-/-^ GC-MSCs with 50 μM DIM to for 48 h, and then collected the CM from these cells. This CM was used to treat gastric cancer cells (HGC-27 and MGC80-3), and the migration ability of the cells was detected using a transwell assay. Treatment with DIM (50μ M) enhanced the migration of gastric cancer cells, which was weakened in β-TrCP^-/-^ GC-MSCs (**Figure 5D**). Furthermore, animal models were used to confirm the proliferation of cancer cells *in vivo*. MGC80-3 pre-cultured with either 0 μM-CM, 50 μM-CM, CM from GC-MSCs transfected with *β-TrCP* siRNA (ctrl+βTRCP^-/-^), or CM from 50 μM DIM treated GC-MSCs transfected with *β-TrCP* siRNA (DIM+β-TrCP^-/-^), were injected subcutaneously  into nude  mice. After 30 days, the tumors were excised (**Figure 5E**), and their weight was evaluated (**Figure 5F**). CM from DIM-treated GC-MSCs significantly increased the tumor weight, and there were no differences between ctrl+β-TRCP^-/-^ and DIM+β-TrCP^-/-^ mice. These results further confirmed that the dosage of DIM that was lethal for gastric cancer cells could not induce cell death in GC-MSCs from TME. Moreover, it promoted the progression of gastric cancer, which was mediated by increased expression of β-TRCP and NF-κB signaling activation to produce more tumor-related cytokines such as CCL-2, IL-6, and IL-8, contributing to the development of gastric cancer (**Figure 6**).

## Discussion

The management of gastric cancer is a tough issue in the medical community because of the difficulty in early diagnosis and its multi-drug resistance (15). Mechanisms such as drug degradation, target site modification, expression of efflux pumps were shown to play important roles in chemotherapeutic failure (16). Even though new anti-cancer drugs and chemotherapies have been developed, there has been no significant improvement in the outcome due to drug resistance, which suggests that the mechanisms underlying this process require urgent investigation. Most studies have focused on the intrinsic or acquired resistance in cancer cells. For example, long non-coding RNA such as lncRNA UCA1 inhibits the apoptosis pathway and induces the resistance in gastric cancer cells to adriamycin and 5-fluorouracil functioning as a sponge of miR-27b to downregulate caspase-3 expression (17). Our group has been trying to discover the mechanism of drug resistance in gastric cancer in a different way by examining the relationship between the chemotherapeutic drugs DIM and mesenchymal stem cells derived from TME, while simultaneously studying the subsequent effects on cancer progression.

DIM is a natural small-molecule anti-cancer drug that has been shown to inhibit cell proliferation and induce cell apoptosis in prostate and breast cancer cells through the regulation of the Akt/FOXO3a/GSK-3/β-catenin/AR signaling axis (18) and reduction of NF-κB activity (19). However, it has also been shown that anti-cancer dosage of DIM can activate autocrine Wnt4 signaling to promote gastric cancer (12). Our previous study indicated that DIM can enhance the stemness of hucMSCs through Wnt/β-catenin signaling (13). MSCs exist in many tissues, including TME, supply necessary nutrition for cancer cells, and have multiple effects on tumors. Taro Ikeda et al. (20) found that bone marrow-derived MSCs can promote gastric cancer by secreting CXCL16 to activate STAT3 and mediate the expression of Ror-1. Our group was the first to isolate GC-MSCs from gastric cancer tissues (8). GC-MSCs share the characteristic of bone marrow-derived MSCs and display unique biomarkers of gastric cancer. Furthermore, GC-MSCs can promote gastric cancer progression by inducing the EMT and triggering M2 macrophage polarization (9, 10). When studying the effects of DIM on MSCs in clinical applications, we should not only consider the safety of DIM with regard to its the lethal effect on cancer cells, but also its action on TME, especially the TME-derived MSCs. Our study found that 50 μM of DIM (corresponding to IC_50_ in gastric cancer cell lines) had almost no lethal effect on GC-MSCs, but could activate the NF-κB signaling pathway and increase the expression of CCL2, IL-6, IL-8, TGF-β. These molecules have been known to promote tumor progression (21).

Paracrine signaling is the most important mechanism by which the MSCs exert biological effects on the target cells. The treatment of gastric cancer lines with conditioned medium of GC-MSCs (22) significantly enhanced the proliferation, invasion, and migration of these cells. These effects could be inhibited by the knockdown of β-TrCP in GC-MSCs. Our study proved that although a regular dosage of DIM could induce cell death in gastric cancer cells, it had a simultaneous effect on MSCs in TME and accelerated the development of tumor cells through paracrine signaling by oncogenic factors. These findings provide a novel direction for a rational usage of anti-cancer drugs and help to establish more effective anti-tumor programs such as adjusting the dosage or combining anti-cancer drugs with inhibitors of the associated signaling pathways, such as NF-κB inhibitors.

In summary, we found that the anti-cancer drugs DIM affected both gastric cancer cells and TME-derived GC-MSCs. The effective dose of DIM reversed the upregulation of the expression of CCL-2, IL-6, and IL-8 in GC-MSCs by β-TrCP-mediated iκBα degradation and NFκB signaling pathway activation, thereby enhancing tumor progression (**Figure 6**). This study results provide a different angle of view on the application of anti-tumor drugs, and imply that a combination of signaling pathway inhibitors with antineoplastic agents may achieve a better curative effect.

## Data Availability Statement

The raw data supporting the conclusions of this article will be made available by the authors, without undue reservation.

## Ethics Statement

The animal study was reviewed and approved by Medical Ethics Committee and Ethics Committee for Experimental Animals of Jiangsu University (IRB protocol number: 2020161).

## Author Contributions

HS, JJ, and HQ conceived the idea and designed the study. HS and YS are responsible for writing the manuscript. All other listed authors participated in the experiments and data collection. All authors contributed to the article and approved the submitted version.

## Funding

This work was supported by the National Natural Science Foundation of China (grant 81972320), Zhenjiang Key Laboratory of High Technology Research on Exosomes Foundation and Transformation Application (grant ss2018003), the Technology Development Project of Jiangsu University (20180361), and the Priority Academic Program Development of Jiangsu Higher Education Institutions (phase III).

## Conflict of Interest

The authors declare that the research was conducted in the absence of any commercial or financial relationships that could be construed as a potential conflict of interest.
