# Supplementary material for: 3,3′-Diindolylmethane Promotes Gastric Cancer Progression via β-TrCP-Mediated NF-κB Activation in Gastric Cancer-Derived MSCs
Source: Front Oncol. 2021 Mar 24;11:603533. doi: 10.3389/fonc.2021.603533 (PMC8024625; doi:10.3389/fonc.2021.603533)
Supplement: Supplementary file 1 [file DataSheet_1.pdf]

3,3'-Diindolylmethane Promotes Gastric Cancer Progression via β-TrCP-Mediated NF-κB activation in Gastric Cancer derived MSCs

Shi Hui[1,2#], Sun Yaoxiang[2, 3#], Ruan Hongru[4], Ji Cheng[1], Zhang Jiahui[1], Wu Peipei[1], Li Linli[1], Huang Chihan[1], Jia Yuanwang[1], Zhang Xu[1], Xu Wenrong[1,2], Jiang Jiajia[1,2*], Qian Hui[1*]

[1]Jiangsu Key Laboratory of Medical Science and Laboratory Medicine, Institute of Stem Cell, School of Medicine, Jiangsu University, Zhenjiang, Jiangsu 212013, China

[2] Institute of Cancer, Affiliated Aoyang hospital of Jiangsu University, Zhangjiagang, Suzhou, 215600, China

[3]Department of Clinical Laboratory, The Affiliated Yixing Hospital of Jiangsu University, Yixing, Jiangsu 214200, China

[4]Department of Burn Orthopedics, The Affiliated People's Hospital, Jiangsu University, Zhenjiang, China.

#These authors contributed equally to this work.

*** Correspondence:**

Qian Hui

lstmmmlst@163.com

Jiang Jiajia

Jiangjiajia_2001@163.com

FIGURE S1

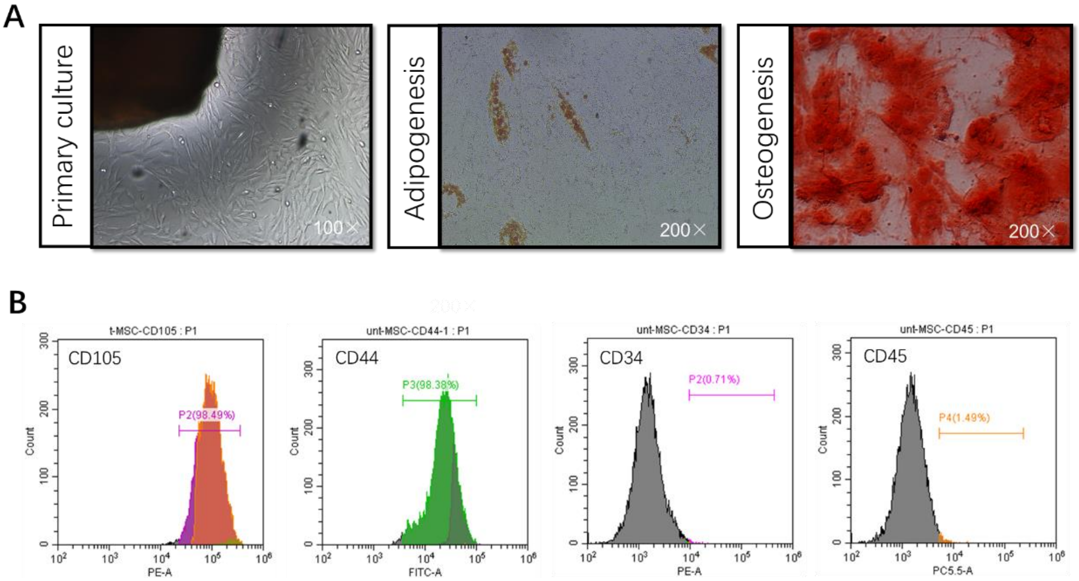

FigureS1. Isolation and identification of GC-MSCs. (A) GC-MSCs were isolated from the gastric cancer tissues through adherent culture. After the initial 1-2 weeks of primary culture, GC-MSCs adhered to the surface of the culture dish and displayed a small population of cells with spindle or fusiform shape. Differentiation capacity of GC-MSCs was evaluated after 16 days of induction in the conditioned media. GC-MSCs presented the ability of differentiating into adipocytes and osteocytes under different inducing conditions, displayed by positive staining of Oil Red O (for adipogenesis) and Alizarin Red S (for osteogenesis). (B) Flow cytometry is used to identify representative markers of GC-MSCs and it turned out to be positive for CD44, CD105 while negative for CD45 and CD34.

FIGURE S2

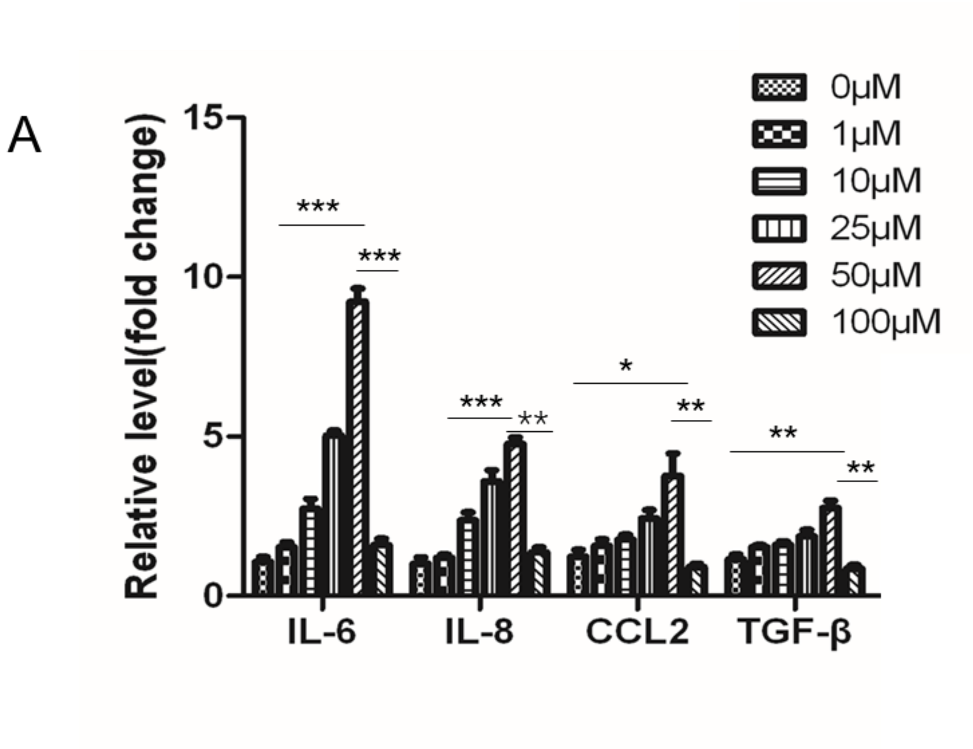

Figure S2. DIM regulate the expression level of cytokines of GC-MSCs. (A) Real-time RT-PCR for the expression of *CCL2*, *IL-6*, *IL-8,* and *TGF-β* genes in GC-MSCs treated with 100 μM DIM for 48 h (n = 3, **P* < 0.05, ** *P* < 0.01, *** *P* < 0.001 compared with the control group).

FIGURE S3

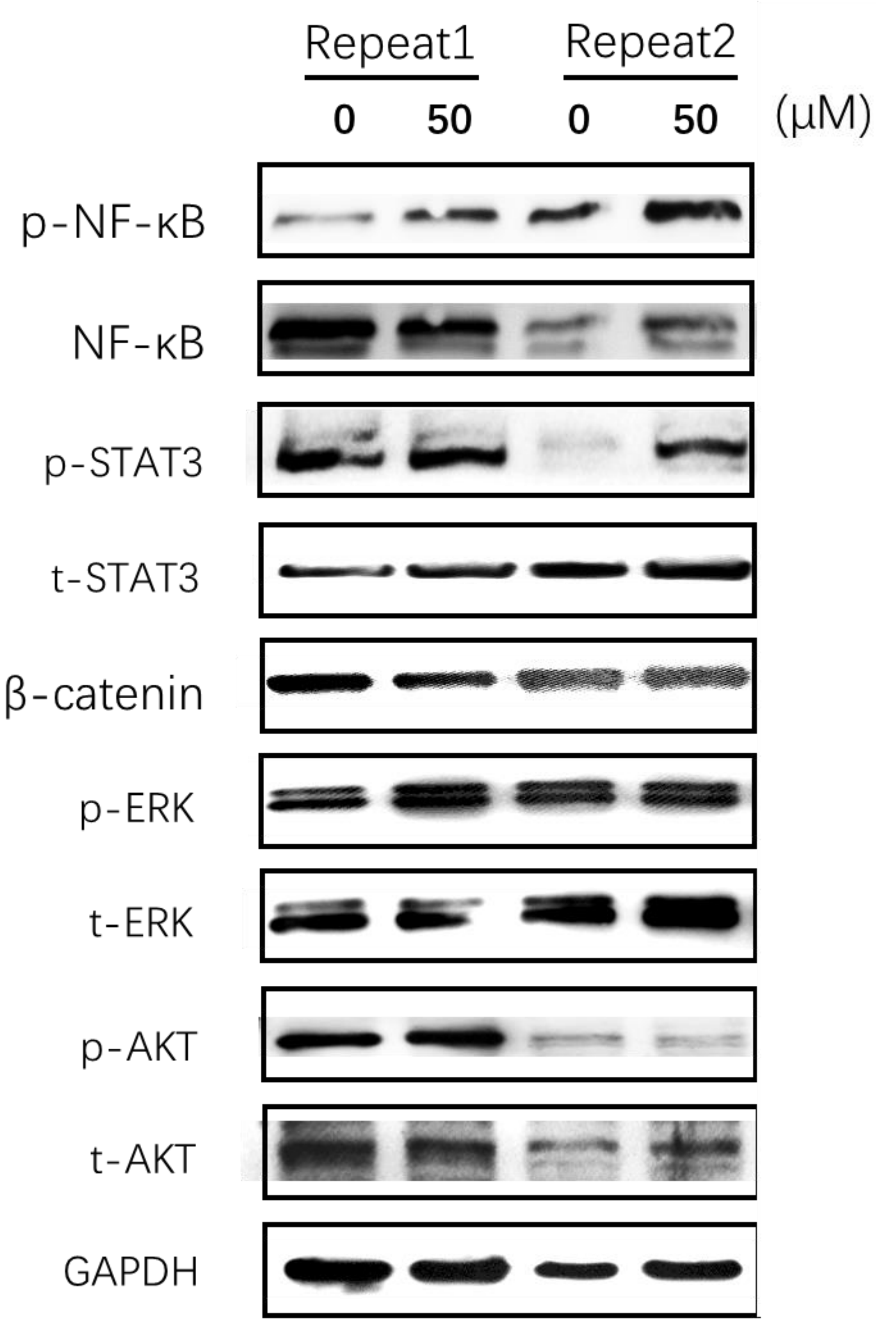

Figure S3. DIM stimulates signaling pathways in different degree. Western for the detection of expression of NF-κB, STAT3, β-catenin, ERK, AKT and GAPDH and in two different batches of GC-MSCs treated with 50 μM DIM for 48 h.

FIGURE S4

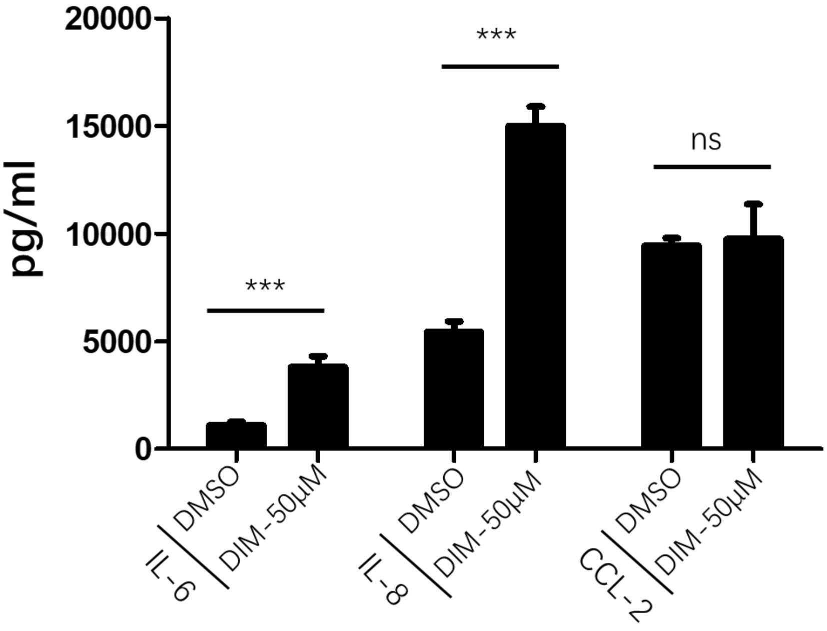

Figure S4. DIM promote the secretion of IL-6, IL-8 and CCL-2 of GC-MSCs. Cytokine profile analysis of GC-MSCs by Luminex immunoassay and the results of IL-6, IL-8 and CCL-2 has been listed. (n=3, *** $P < 0.001$ compared with the control group).
